# The Anxiety and Depression in Rheumatoid Arthritis Patients Treating with Disease-Modifying Anti-Rheumatic Drugs during the COVID-19 Pandemic

**DOI:** 10.31138/mjr.33.4.430

**Published:** 2022-12-31

**Authors:** Gökhan Sargın, Huseyin Baygin, Taskin Senturk

**Affiliations:** Department of Rheumatology, Aydın Adnan Menderes University, Aydın, Turkey

**Keywords:** rheumatoid arthritis, COVID-19, anxiety, depression, disease-modifying anti-rheumatic drugs

## Abstract

**Objective:**

Anxiety and depression are associated with the risk of illness, presence of physical symptoms, and poor health in the COVID-19 epidemic. Our aim is to assess the severity of anxiety and depression in rheumatoid arthritis (RA) patients treated with disease-modifying anti-rheumatic drugs during the COVID-19 pandemic.

**Material and methods:**

The study is a longitudinal, hospital-based survey study including 102 RA patients receiving disease-modifying anti-rheumatic drugs with a mean of 55,2±11,9 years. Demographic data, educational status, marital status, employment status, economic status, patients with psychiatric disorders (with the use of prescribed medication for treatment), and medications were recorded. The severity of depression and anxiety were evaluated with the Beck Anxiety and Depression Inventory at the first and second visit of the follow-up during the pandemic period.

**Results:**

The mean Beck depression inventory score was found to be higher in the conventional synthetic DMARDs group than in biological DMARDs (12,1±8,2 vs 11,6±9,2, p=0,554). 46 (65,7%) had mild to severe anxiety symptoms in RA patients treated with conventional synthetic DMARDs, on the first visit. There was no significant difference in anxiety and depression status between the first and second visits. The difference in anxiety and depression symptoms between RA patients receiving conventional synthetic and biological DMARDs does not attain statistical significance. Also, no significant differences were found in anxiety and depression scores in the comparisons for gender, education, marital, working, and economic status.

**Conclusions:**

The severity of depression and anxiety were higher in RA patients receiving conventional synthetic DMARDs and biological DMARDs during the COVID-19 pandemic. Also, RA patients are likely to experience anxiety and depression during the period of the pandemic.

## INTRODUCTION

In late December 2019, a novel coronavirus called SARS-CoViD-2 (Severe Acute Respiratory Syndrome Coronavirus-2) was identified series of pneumonia cases in Wuhan City, China. This virus has spread all over the world, causing a global epidemic.^1^ SARS-CoV-2 is an enveloped positive-strand RNA virus that is transmitted via respiratory droplets of infected patients or vertically to the fetus during pregnancy. Clinical manifestations can range from asymptomatic disease to fever, fatigue, myalgia, dry cough, dyspnea, and respiratory failure.^1^ Pneumonia, acute respiratory distress syndrome, sepsis, cardiac complications, and secondary infections are among the important causes of morbidity and mortality in SARS-CoViD-2.^1,2^ Other factors affecting morbidity and mortality in viral infections were reported as mood disorders, anxiety, post-traumatic stress syndrome, panic disorder, and bipolar disease.^3^ Depression and psychological stress decrease cell-mediated immunity and increase inflammatory processes during infections.^3^

Viral diseases are included in the etiology of some neuropsychiatric disorders.^4^ Patients with hepatitis C virus (HCV) have fatigue, depression, and cognitive dysfunction.^4^ In addition, depression, anxiety, sleep disorders, psychosis, and delirium have been associated with pegylated interferon alfa-2a treatment in patients with HCV.^4^ Depression, anxiety, delirium, dementia, and manic-spectrum disorders have also been reported in patients with human immunodeficiency virus depending on the disease itself or the agents used in its treatment.^4^ The SARS outbreak in 2003 led to general stress, post-traumatic stress syndrome, and depressive disorder directly or indirectly in infected patients.^5,6^ In the SARS epidemic, 35% of patients had “moderate to severe” or “severe” and/or depressive symptoms after 1 month of recovery.^7^ The disease itself, the uncertainty about the efficacy of treatment, negative news in the media, the quarantine environment, the concern of infecting their loved ones, and high death rates cause increased stress in patients.^8^ In addition, the economic impact also contributes to depressive symptoms and levels.^5^ The medical history for psychiatric illness, loss of family members in the epidemic, and the sense of stigma associated with the disease were thought to be associated with neuropsychiatric problems in the Middle East respiratory syndrome outbreak.^9^

Anxiety and depression are associated with the risk of illness, presence of physical symptoms, and poor health in the COVID-19 epidemic.^10^ Also, not having enough surgical masks and being uncomfortable working from home are associated with poor mental health.^11^ Post-traumatic stress syndrome, depression, and anxiety have been reported in COVID-19 patients even after hospitalisation. It has been thought that these conditions occur due to the immune response to the virus, social isolation, the psychological impact of a new severe and potentially mortal disease, the anxiety of contagion to others, and stigmatisation.^12^ Factors such as difficulties in daily life, self-confidence in coping with stress, and expected health status are associated with depression in rheumatoid arthritis (RA) patients.^13^ In another study, there has been reported a relationship between poor sleep quality and anxiety, depression, self-efficacy, and stigma in RA patients.^14^ Here, sleep disturbance was found to be positively correlated with age, pain, disease activity, depression, anxiety, and stigma, while self-efficacy was negatively correlated with sleep disturbance.^14^

In a cross-sectional study, it has been determined that depression is associated with social determinants of health, such as food insecurity in RA patients.^15^ RA patients treated with immunosuppressive drugs during the pandemic are at risk for anxiety and depression.

In this study, we aim to evaluate the changes in the severity of depression and anxiety in patients with rheumatoid arthritis treated with disease-modifying anti-rheumatic drugs during the COVID-19 pandemic.

## MATERIAL AND METHODS

The study is a longitudinal, hospital-based survey study including 102 rheumatoid arthritis patients. All patients aged 18 years and older included in the study were diagnosed with RA according to the 2010 American College of Rheumatology classification criteria.^16^ We excluded patients with any connective tissue disease other than RA, infections, and hematological and solid malignancies were excluded from the study. Age, gender, disease duration, disease activity score-28 (DAS-28), educational status, marital status, employment status, economic status, comorbidities, patients with psychiatric disorders (with the use of prescribed medication for treatment), and rheumatological medication (conventional synthetic or biological disease-modifying anti-rheumatic drugs [DMARDs]) of the patients were recorded. The study protocol was approved by the Faculty of Medicine Ethics Committee and designed consistent with the Declaration of Helsinki (a consent number and date of receipt: E-53043469-050.04.04-40995, 2021/97).

Beck Depression Inventory consists of 21 items to evaluate and measure the severity of depression, and each item indicates a behavioral situation specific to depression. Items consisting of 4 sentences graded from low to high (0–3 points) are evaluated as follows: 0–9 as no/minimal, 10–18 as mild, 19–29 as moderate, and 30–63 as severe depression. _17_ The validity and reliability of the Beck Depression Inventory for the Turkish population were tested by Hisli.^18^ Beck Anxiety Inventory consists of 21 self-reported items rated from 0 to 3 to assess the anxiety during the past week. The total score varies between 0 and 63 and indicates different severity degrees of depression (0–7 point: no/low, 8–15 point: mild, 16–25 point: moderate, 26–63 point: severe).^19^ Ulusoy et al. validated the Turkish version of the Beck Anxiety Inventory.^20^

### Statistical analysis

Statistical analyses were performed with Statistical Package for Social Sciences (SPSS) for Windows (SPSS version 21.0, IBM, USA). The data were presented as mean±standard deviation, median (25–75 percentile), frequency (n), and percentages (%). Categorical variables were analyzed by using Pearson’s chi-square test and Fisher’s exact test. A Kolmogorov-Smirnov test was used to determine the homogeneity of the data. Continuous variables were analyzed by using the Mann-Whitney U test and Independent-samples T-test according to the distribution. The Wilcoxon signed-rank test was used to compare two related samples. Multivariable regression analysis was performed to correlate demographic data with anxiety and depression. Regression analysis was performed for follow-up anxiety and depression symptoms as dependent variables and demographic data, duration between the two assessments and baseline anxiety and depressive data as independent variables. P-values of less than 0.05 were considered statistically significant.

## RESULTS

A total of 102 patients, including 24 male (23,5%) and 78 female (76,5%) were included in the study. The mean age of the patients was 55,2±11,9 years (19–75 years) and the mean DAS-28 was 4,0±0,9 in patients treating with conventional synthetic DMARDs and 3,8±0,9 for biological DMARDs group. The mean Beck Anxiety Inventory score of the patients who were treated with conventional synthetic DMARDs was 13,1±11,6. The mean score was higher than in RA patients treated with biological DMARDs. The mean Beck depression inventory score was higher in the conventional synthetic DMARDs group than biological DMARDs (12,1±8,2 vs 11,6±9,2). However, no significant differences were found in anxiety and depression scores between the two groups. Also, there were no statistically significant differences in the Beck Anxiety Inventory and Beck Depression Inventory scores in the comparison of gender, education status, marital status, employment status, and economic status of all patients. Demographic characteristics, anxiety, and depression scores of the RA patients are shown in **Table 1**.

**Table 1.** Demographic characteristics, anxiety, and depression scores of patients with rheumatoid arthritis.

	**Conventional synthetic DMARDs (n=70)**	**Biological DMARDs (n=32)**	**p-value**

**Age, years**	55,8±11,4	53,9±13,2	0,670

**Gender, n (%)**			
• female	52 (74,3)	26 (81,3)	
• male	18 (25,7)	6 (18,7)	0,442

**Disease Activity Score-28, mean±SD**	4,0±0,9	3,8±0,9	0,161

**Disease Duration, years, mean±SD**	6,3±3,8	6,8±3,6	0,341

**Comorbidity, n (%)**			
• diabetes mellitus	13 (18,6)	7 (21,9)	0,697
• hypertension	35 (50)	12 (37,5)	0,240
• coronary artery disease	7 (10)	3 (9,4)	0,922
• chronic heart failure	1 (1,4)	1 (3,1)	0,566

**Education Status, n (%)**			
• less than high school	54 (77,1)	28 (87,5)	
• high school	11 (15,7)	2 (6,3)	0,431
• university/postgraduate	5 (7,1)	2 (6,3)	

**Marital Status, n (%)**			
• married	57 (81,4)	26 (81,3)	
• single	13 (18,6)	6 (18,7)	0,983

**Working Status, n (%)**			
• working	11 (15,7)	8 (25,0)	
• not working	59 (84,3)	24 (75,0)	0,264

**Economic Situation, n (%)**			
• less than my expenses	35 (50,0)	18 (56,3)	
• equal to my expenses	32 (45,7)	12 (37,5)	
• more than my expenses	3 (4,3)	2 (6,3)	0,670

**Psychiatric Disorders, n (%)**			
• yes	11 (15,7)	5 (15,6)	
• no	59 (84,3)	27 (84,4)	0,991

**Beck Anxiety Inventory Score, mean±SD**			
• 1st visit	13,1±11,6	12,6±11,4	0,770
• 2nd visit	12,5±11,2	12,7±11,9	0,865

**Beck Depression Inventory Score, mean±SD**			
• 1st visit	12,1±8,2	11,6±9,2	0,554
• 2nd visit	11,5±8,8	11,7±9,2	0,983

All RA patients completed the questionnaires at the first and second visits during the pandemic period. The mean follow-up between the first visit to the second visit was 12,6±3,9 weeks for conventional synthetic DMARDs and 10,4±3,7 weeks for biological DMARDs. According to the Beck Depression Inventory score, 46 (65.7%) had mild to severe anxiety symptoms and 42 (60%) had mild to severe depression symptoms in RA patients treating with conventional synthetic DMARDs, on the first visit. At the second visit, 45 (64.2%) of the patients had anxiety symptoms and 38 (54.2%) had depression symptoms. According to the Beck Depression Inventory score, 46 (65,7%) had mild to severe anxiety symptoms and 42 (60%) had mild to severe depression symptoms in RA patients treating with conventional synthetic DMARDs on the first visit.

At the second visit, 45 (64.2%) of the patients had anxiety while 38 (54.2%) had depression. The anxiety levels of the biological DMARDs group at the first visit were as mild, moderate, and severe in 10 (31,3%), 2 (6,3%), and 6 (18,8%) patients, respectively. At the second visit, the rates were 10 (35.7%), 1 (17.1%) and 7 (11.4%), respectively. The severe depression ratio was higher in RA patients receiving biological DMARDs compared to conventional synthetic DMARDs (6,3% vs 2,9%). There was no significant difference in anxiety and depression between the first and second visits. Also, no significant differences were found in depression and anxiety status between both conventional and biological agent groups. The distribution of the Beck Anxiety Inventory score and Beck Depression Inventory score are shown in **Table 2** and **Table 3**.

**Table 2.**
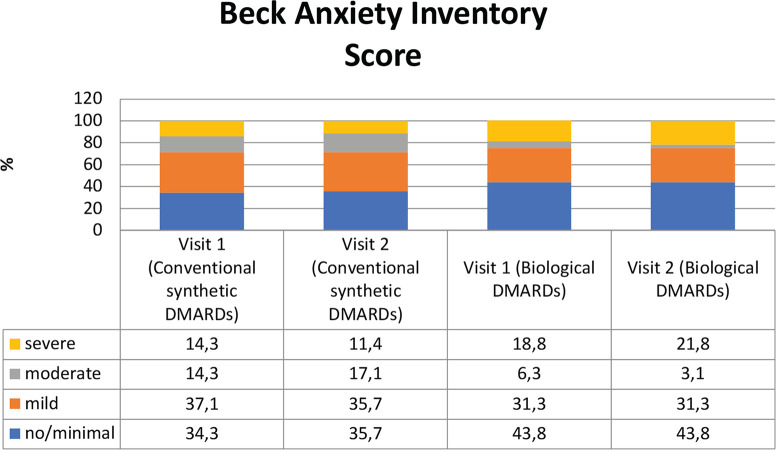
The distribution of the Beck Anxiety Inventory score in RA patients receiving conventional synthetic DMARDs and biological DMARDs at visit 1 and visit 2.

**Table 3.**
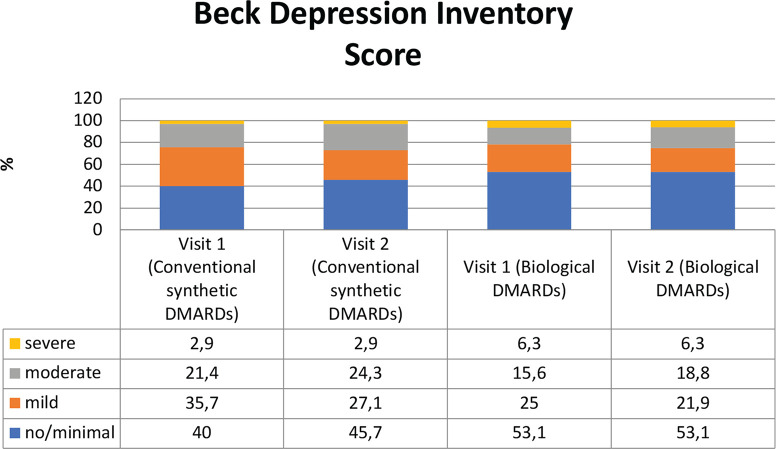
The distribution of the Beck Depression Inventory score in RA patients receiving conventional synthetic DMARDs and biological DMARDs at visit 1 and visit 2.

In the conventional synthetic DMARDs group, the mean Beck Anxiety Inventory scores were 13,1±11,6 at the first visit and 12,5±11,2 at the second visit. The ratios were 12,6±11,4 and 12,7±11,9 for the biological DMARDs group, respectively. The mean Beck Anxiety Inventory score and Beck Depression Inventory score were found to be decreased at the second visit compared to the first visit for both the traditional synthetic and biological DMARDs group. However, no significant differences were found in scores between visits. Positive correlation was found between DAS-28 and Beck Anxiety Inventory score (r=0,737, p<0,001) and Beck Depression Inventory scores (r=0,456, p<0,001). In the multivariable regression, higher Beck Anxiety Inventory score and Beck Depression Inventory scores at the first visit, and DAS-28 were independent risk factors for anxiety and depression at follow-up in both treatment groups.

## DISCUSSION

In the study, we aimed to evaluate the changes in the severity of anxiety and depression in patients with RA treating with Disease-Modifying Anti-Rheumatic Drugs during the COVID-19 pandemic. The mean Beck Anxiety Inventory score was higher than those who were treated with biological DMARDs compared to conventional synthetic DMARDs. The mean Beck Depression Inventory score was higher in the conventional DMARDs group than biological DMARDs. The mean Beck Anxiety Inventory score and Beck Depression Inventory score were found to be decreased at the second visit compared to the first visit for both DMARDs group. There was no statistically significant difference in Beck Anxiety Inventory score and Beck Depression Inventory score for gender, marital, employment, economic and educational status in all RA patients.

During the pandemic process, the severity of containment and closure measures should be taken into account at the point in time for each participant’s assessments. In this context, the Oxford COVID-19 Government Response Tracker (OxCGRT), a dataset, constantly updated, expanding, and easy to use with global scope provide to detection of COVID-19 cases, deaths, and their effects on economic and social welfare [21]. Anxiety and depression have been reported as common psychological problems during crisis periods in pandemics. Higher depressive levels were found in individuals with quarantined or indirectly exposed to SARS in a study to investigate the psychological effects of the SARS epidemic in 2003.^5^ The prevelance of depressive symptoms experienced in the previous week in the participants included in the study was 3.7%.^5^ In another study that investigated the psychological effects of the SARS epidemic, the prevalence of symptoms of depression, anxiety, and post-traumatic stress disorders was reported to be between 10% to 18%.^22^ In a survey-study conducted during the initial phase of COVID-19 pandemic, it was reported that 28.8% of the participants showed moderate to severe anxiety symptoms and 16.5% moderate to severe depressive symptoms.^10^ Female gender, student status, the presence of specific physical symptoms such as myalgia, and dizziness were associated with higher levels of stress, anxiety, and depression.^10^

The prevalence of depression in RA patients was reported to be between 14.8% and 38.8% according to different measurement methods.^23^ There has been a significant increase in suspected depression after the COVID-19 pandemic. In a study, depression was reported as 10% in RA patients during the COVID-19 pandemic.^24^ While the definite anxiety rate was approximately 9% in RA patients before the pandemic, this rate increased to 12% in 2020 during the pandemic period.^24^ In another study investigating the psychological effects of the pandemic on patients with rheumatic diseases, the frequency of anxiety was found to be 20%, depression 43%, and post-traumatic stress 28%.^25^ Female gender, having a lower education level, having children, and living in a large family are independent risk factors associated with anxiety and depression in patients with the rheumatic disease.^25^ RA patients are more likely to experience anxiety symptoms due to higher infection risk than the healthy population.^22^ The confusing information about the benefits and harms of rheumatic drugs against COVID-19 may contribute to this situation.^24^ In our study, RA patients treated with conventional synthetic DMARDs had mild to severe anxiety symptoms in 46 (65.7%) and mild to severe depression symptoms in 42 (60%) at the first visit, based on the Beck Depression Inventory. At the second visit, 38 (54,2%) of the patients showed depressive symptoms. The mean Beck Anxiety Inventory score and Beck Depression Inventory score were decreased at the second visit compared to the first visit for both conventional synthetic and biological DMARDs group. However, no significant differences were found in scores between visits. In addition, there were no statistically significant differences in the Beck Anxiety Inventory and the Beck Depression Inventory scores in the comparisons of all patients for gender, education, marital, working, and economic status.

There has been occurred a concern associated with the use of immunosuppressive drugs in patients with the rheumatological disease with the COVID-19 pandemic. EULAR recommends that patients with rheumatic diseases taking conventional/biological DMARDs, JAK inhibitors, steroids, and methotrexate should not discontinue unless a specific cause.^26^ Discontinuation of the drugs may cause increased disease activity, morbidity, and mortality. In a study, it was determined that 22.4% of the patients with rheumatic diseases discontinued or stopped their medications during the COVID-19 pandemic. And most of them were biological DMARDs such as anti-IL-1/anti-TNF agents, tocilizumab, and rituximab.^25^

In our study, there was no patient who discontinued both conventional synthetic and biological DMARDs, except for patients with a history of coronavirus. The limitation of the study was that it was a short-term evaluation for the depression and anxiety severity during the COVID-19 pandemic.

## CONCLUSIONS

Based on the results of this study, the severity of anxiety and depression was higher in RA patients taking immunosuppressive therapy during the COVID-19 pandemic compared to the general population. The difference in anxiety and depression symptoms between RA patients receiving conventional synthetic and biological DMARDs does not attain statistical significance. RA patients are likely to experience anxiety and depression during the period of the pandemic. Therefore, it is necessary to be careful in terms of psychological changes and to take precautions if necessary. The anxiety and depression rates have not changed statistically in a short time in RA patients. This may be related to the fact that even during the deepening of the pandemic crisis, according to the recommendations of rheumatology societies and especially EULAR, it was stated that drugs such as conventional/biological DMARDs, JAK inhibitors, steroids, and methotrexate should not be discontinued unless COVID-19 infection. However, studies with longer follow-up periods are needed for long-term data.
